# Comparative effectiveness of budesonide EC and telitacicept in proteinuria and eGFR trajectories in IgA nephropathy: a retrospective cohort study

**DOI:** 10.3389/fimmu.2026.1821774

**Published:** 2026-05-13

**Authors:** Xingsheng Zuo, Yaqin Wang, Fangfang Ma, Xianyuan Zhu, Chenglong Zhao

**Affiliations:** 1 Department of Pharmacy, Henan Provincial People’s Hospital, People’s Hospital of Zhengzhou University, Zhengzhou, Henan, China; 2 School of Pharmacy, Henan University of Chinese Medicine, Zhengzhou, Henan, China

**Keywords:** budesonide, comparative effectiveness, EGFR, IgA nephropathy, proteinuria, telitacicept

## Abstract

**Background:**

IgA nephropathy (IgAN) is the most common primary glomerulonephritis worldwide. However, the lack of direct comparative evidence between the emerging targeted therapies, budesonide enteric-coated (EC) capsule and telitacicept, creates uncertainty in clinical decision-making. This study aimed to conduct a head-to-head comparison of their efficacy in reducing proteinuria and preserving renal function, and to explore efficacy stratification based on baseline characteristics.

**Methods:**

We conducted a single-center retrospective cohort study at Henan Provincial People’s Hospital, including 95 adults with biopsy-proven IgAN who initiated budesonide EC (n ;= ;46) or telitacicept (n ;= ;49) from January 2022 to December 2025. We assessed 6-month changes in 24-hour proteinuria and eGFR using linear mixed-effects models adjusted for prespecified baseline covariates—age, sex, time from biopsy to treatment, baseline proteinuria and eGFR, comorbidities, and concomitant medications. Findings were evaluated in six sensitivity analyses.

**Results:**

In this selected cohort of 95 patients with biopsy-proven IgAN, the effect of treatment on proteinuria reduction was modified by baseline proteinuria level (interaction P = 0.003), with greater relative benefit of telitacicept observed at mild-to-moderate baseline proteinuria. This interaction remained significant in a propensity score–matched (PSM) analysis (p = 0.003). Budesonide EC was associated with a higher mean eGFR during follow-up in the primary model (β = 4.090 mL/min/1.73 m²; p = 0.018), though this association attenuated and lost statistical significance in the matched cohort (β = 5.022 mL/min/1.73 m²; p = 0.085).

**Conclusions:**

The relative antiproteinuric effect of telitacicept versus budesonide EC may be modified by baseline proteinuria, with a possible trend toward greater benefit in patients with mild-to-moderate levels. In contrast, although budesonide EC was associated with higher mean eGFR during follow-up in the primary analysis, this difference attenuated after PSM and likely reflects baseline imbalance rather than a true differential effect on renal trajectory. These observations are limited by the retrospective design and residual confounding, and require confirmation in larger prospective studies.

## Introduction

IgA nephropathy (IgAN), the most common primary glomerulonephritis globally, exhibits a distinct East-West epidemiologic gradient, being most prevalent in East Asia where it accounts for 40–50% of native kidney biopsies, compared to 10–20% in North America, up to 20% in Europe, and less than 5% in Africa (1–3). It is a progressive disease and a leading cause of end-stage kidney disease worldwide. Approximately 20–40% of patients with persistent proteinuria progress to kidney failure within 10–20 years of diagnosis (4–6). For decades, management has relied on supportive therapy with renin–angiotensin system inhibitors, while corticosteroid use has been limited by substantial toxicity despite a modest antiproteinuric effect, as demonstrated in the TESTING trial (7, 8). This clear unmet need has driven the development of novel, pathogenesis-targeted therapies (9).

Two targeted therapies, acting through distinct mechanisms, are now in clinical use for IgAN: budesonide EC, a gut-selective corticosteroid that reduces production of galactose-deficient IgA1(Gd-IgA1), and telitacicept, a recombinant fusion protein that inhibits the BAFF/APRIL axis to curb autoantibody formation (10, 11). Although randomized controlled trials (RCTs) have established the efficacy of each agent in reducing proteinuria, direct head-to-head comparative data are lacking (12–14). This gap leaves clinicians without high-level evidence to guide personalized choice between these mechanistically divergent options—a key clinical dilemma given their distinct profiles (15). To address this, we conducted the first real-world comparative effectiveness study of budesonide EC versus telitacicept. Our objectives were to compare their effects on proteinuria reduction and estimated glomerular filtration rate (eGFR) preservation over 6 months, and to assess whether baseline proteinuria modifies the treatment response, which may inform patient stratification (16, 17).

The efficacy of budesonide EC was established in the pivotal phase 3 NefIgArd trial, which demonstrated a 27% greater reduction in proteinuria at 9 months compared with placebo, alongside a trend toward eGFR stabilization over 2 years (13). This agent exerts its effect by homing to the distal ileum to suppress the proliferation and maturation of Gd-IgA1–producing B cells in Peyer’s patches, thereby targeting the gut mucosal origin of IgAN (18). In contrast, telitacicept—a recombinant fusion protein that dual-targets the B-cell survival cytokines BAFF and APRIL—reduced mean proteinuria by 49% from baseline in Chinese phase 2/3 trials (11, 19). Both programs primarily enrolled patients with moderate proteinuria, with median baseline levels of approximately 1.6 to 1.8 g/24h (interquartile ranges: 1.29–2.41 for telitacicept; 1.24–2.49 for budesonide EC). By design, these trials excluded individuals at the extremes of the disease spectrum—specifically, those with very low eGFR (e.g., <30 mL/min/1.73 m^2^) or severe proteinuria (>3.5 g/24h), thresholds commonly applied in pivotal IgAN studies. Consequently, while providing robust evidence of efficacy within a defined patient profile, these trials generated no direct comparative data between the two agents and offer limited insight into treatment response among patients with the most severe proteinuric presentations (20).

This evidence gap raises an important, unresolved clinical question: in real-world practice, can readily available characteristics—particularly baseline proteinuria, a cardinal prognostic marker and primary treatment target—guide the choice between these two mechanistically distinct first-line options (21–26)? To answer this, we conducted a real-world cohort study with two specific aims: (1) to compare the effectiveness of budesonide EC and telitacicept with respect to proteinuria reduction and eGFR preservation over 6 months; and (2) to assess whether baseline proteinuria modifies the relative antiproteinuric effect of the two agents.

## Methods

### Study design and population

This retrospective, single-center cohort study was conducted at Henan Provincial People’s Hospital (Zhengzhou, China). We identified all consecutive adults (aged ≥18 years) with biopsy-confirmed IgAN who initiated treatment with either budesonide EC or telitacicept during routine clinical care between January 2022 and December 2025. Eligible patients were required to have baseline measurements of both proteinuria and eGFR, as well as at least one post-baseline assessment within six months of treatment initiation. A total of 95 patients met these criteria and formed the analytic cohort. Of these, complete Oxford-MEST-C scores (2016 revision) were available for 76 patients (80%) and are presented in **Supplementary Table 2**.

This study was conducted in accordance with the Declaration of Helsinki. The protocol was approved by the Hospital Ethics Committee (Approval No. 2025-223), and written informed consent was obtained from all participants.

### Exposure and covariates

The primary exposure was initiation of treatment with either budesonide EC (16 mg once daily) or telitacicept (160 mg subcutaneously once weekly), as prescribed in routine practice. Treatment choice was at the discretion of the attending nephrologist based on routine clinical practice.

Covariates for adjustment were prespecified based on their established prognostic relevance in IgAN and baseline distribution. These included demographic factors (age, sex), time from diagnostic kidney biopsy to initiation of study treatment (in months), baseline disease severity (proteinuria [g/24h] and eGFR [mL/min/1.73 m²]), key comorbidities (hypertension, diabetes), and concomitant medications with potential nephroprotective or immunosuppressive effects (renin–angiotensin system inhibitors, sodium–glucose cotransporter-2 inhibitors, glucocorticoids, and mycophenolate mofetil). Comorbidities and concomitant medications were modeled as dichotomous (yes/no) variables.

### Outcome measures

The primary outcomes were changes in 24-hour proteinuria (g/24h) and eGFR (mL/min/1.73 m²) over the first six months of treatment. All available post-baseline measurements from monthly clinic visits, scheduled within a ±14-day window of each nominal month, were included in the analysis. No imputation was performed for missing outcome values. eGFR was calculated using the Chronic Kidney Disease Epidemiology Collaboration (CKD-EPI) creatinine equation (27).

### Statistical analysis

Longitudinal changes in proteinuria and eGFR were analyzed using linear mixed-effects models. Follow-up visits were scheduled monthly over six months, with actual dates allowed within a ±14-day window of each nominal month. Time was modeled as a continuous variable (months from treatment initiation) using exact visit intervals. Patients contributed 2–7 measurements (median: 5), reflecting real-world follow-up variation. To account for within-individual correlation and heterogeneous trajectories, models included participant-specific random intercepts and random slopes for time. A first-order autoregressive [AR (1)] covariance structure for the within-subject residuals was selected based on Akaike (AIC) and Bayesian (BIC) criteria and estimated via restricted maximum likelihood (REML). All available data were used under the assumption that missing outcome values were missing at random (MAR). This is consistent with likelihood-based mixed models, which provide unbiased estimates under MAR when the model is correctly specified (28).

All prespecified covariates were considered in model development. Candidate models were compared using AIC and BIC information criteria, and the model with optimal fit and clinical interpretability was selected as the primary analysis model.

Prespecified interactions between treatment and (1) time and (2) baseline proteinuria were tested in the primary model. Given that the interaction with baseline proteinuria was statistically significant and robust to sensitivity analyses—including propensity score matching (PSM)—a Johnson–Neyman procedure was applied to characterize the range of baseline proteinuria values over which the treatment difference was statistically significant. Results were visualized using the interactions package in R (v4.4.3). No significant interactions were observed for eGFR; thus, moderation analysis was restricted to proteinuria.

### Sensitivity and robustness analyses

We conducted six prespecified sensitivity analyses to assess robustness:

1. A reduced model excluding baseline covariates with p < 0.10 that were not considered clinically essential;
2. Additional adjustment for baseline renal parameters (proteinuria, eGFR) and comorbidities (hypertension, diabetes);
3. A fully adjusted model including all measured baseline variables;
4. Exclusion of patients with extreme standardized residuals (|ZRE| > 3);
5. Re-estimation using a compound symmetry covariance structure;
6. PSM on imbalanced baseline covariates (**Table 1**), yielding 24 matched pairs (n = 48).

**Table 1:** Baseline characteristics of patients by treatment group.

Variable	Budesonide EC (n=46)	Telitacicept(n=49)	P value
Demographics			
Age, years	44.1 ± 14.7	37.2 ± 12.9	0.016
Male sex, n (%)	31 (67.4%)	27 (55.1%)	0.220
Clinical Characteristics			
Time from biopsy to treatment, months	1.1 (0.7–3.2)	0.5 (0.2–1.1)	<0.001
Hypertension, n (%)	29 (63.0%)	29 (59.2%)	0.700
Diabetes, n (%)	2 (4.3%)	5 (10.2%)	0.437
Laboratory Measures			
Baseline proteinuria, (g/24h)	1.50 (0.65–2.58)	3.48 (1.96–4.87)	0.006
Baseline eGFR, mL/min/1.73m²	76.1 ± 33.1	66.7 ± 33.0	0.167
Concomitant Medications at Baseline			
Glucocorticoids use, n (%)	5 (10.9%)	25 (51.0%)	<0.001
MMF Use, n (%)	6 (13.0%)	10 (20.4%)	0.338
RASi Use, n (%)	39 (84.8%)	35 (71.4%)	0.117
SGLT2i Use, n (%)	15 (32.6%)	12 (24.5%)	0.381

Data are presented as mean ± SD, median [interquartile range], or number (%).

Baseline, time of treatment initiation.

eGFR estimated using CKD-EPI equation.

P-values compare budesonide EC vs telitacicept groups.

SGLT2i, sodium-glucose cotransporter-2 inhibitor.

RASi, renin-angiotensin system inhibitor.

MMF, mycophenolate mofetil,.

All sensitivity analyses applied the same modeling framework as the primary analysis. Primary analyses were performed in SPSS (v26; IBM Corp.), with statistical significance defined as two-sided p < 0.05. Graphical outputs were generated in R.

## Results

### Overall population characteristics

**Figure 1:**
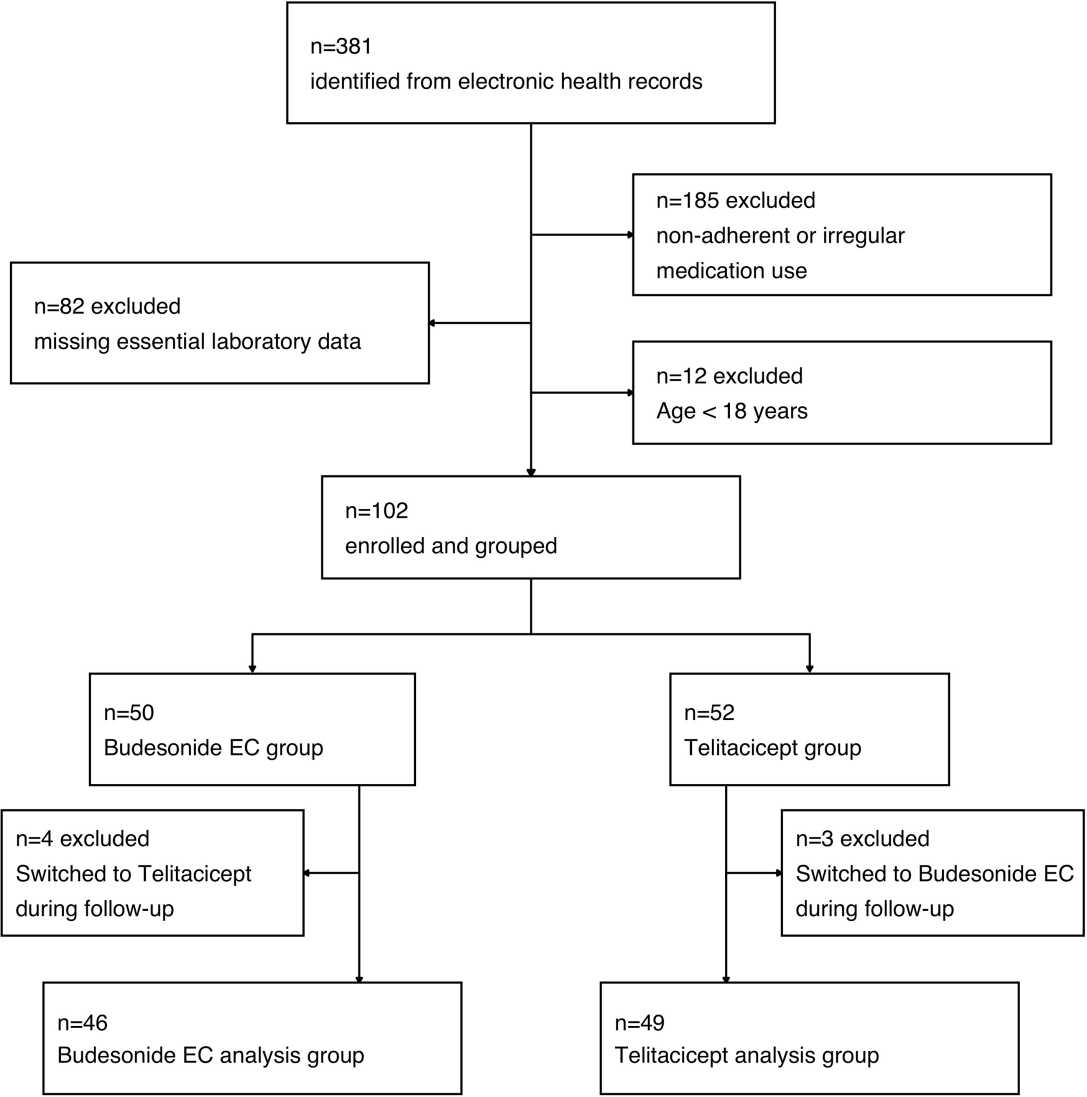
Study flowchart. Excluded for missing essential laboratory data (n = 82): patients lacking baseline 24-hour proteinuria or baseline eGFR, or with no follow-up measurements at any time after baseline.

We identified 381 consecutive adults with biopsy-proven IgAN from electronic health records between January 2022 and December 2025. After applying the inclusion criteria, 95 patients comprised the analytic cohort (**Figure 1**): 46 initiated budesonide EC and 49 telitacicept.

Baseline characteristics are summarized in **Table 1**. Patients receiving telitacicept were younger (mean age, 37.2 vs. 44.1 years; p = 0.016), had a shorter interval from biopsy to treatment initiation (median, 0.5 vs. 1.1 months; p < 0.001), and presented with higher baseline proteinuria (median, 3.48 vs. 1.50 g/24h; p = 0.006) than those receiving budesonide EC. Concomitant use of low-dose glucocorticoids (prednisone ≤10 mg/day) was also more frequent in the telitacicept group (51.0% vs. 10.9%; p < 0.001). No significant between-group differences were observed in sex, prevalence of hypertension or diabetes, baseline eGFR, or use of other background nephroprotective or immunosuppressive medications.

The primary analytic model included all prespecified prognostic covariates; its structure was finalized based on AIC/BIC criteria and clinical plausibility.

### Longitudinal changes in proteinuria: effect modification by baseline proteinuria

Analysis of the linear mixed-effects model revealed a significant interaction between treatment group and baseline proteinuria (β = –0.275 per g/24h; 95% CI, –0.456 – –0.094; p = 0.003; **Table 2**), indicating that the effect of treatment on proteinuria reduction varied by baseline severity. In contrast, the apparent difference in proteinuria decline rates between groups over time (treatment group × treatment time: β = 0.244 g/24h per month; 95% CI, 0.120–0.367; p < 0.001) was not robust to PSM, where this interaction became non-significant (p = 0.919; **Supplementary Table 1**).

**Table 2:** Effect modification of treatment on proteinuria trajectory by time and baseline proteinuria: results from the linear mixed-effects model.

Variable	Comparison	Estimate (β)	95% CI	p-value
Fixed Effects				
Intercept	—	0.103	(-0.845, 1.052)	0.830
Sex	Female vs. Male (reference)	0.313	(-0.130, 0.755)	0.164
Age	Per year increase	0.001	(-0.015, 0.017)	0.889
Treatment Group	Budesonide EC vs. Telitacicept (reference)	0.407	(-0.332, 1.146)	0.278
Treatment Time	Per month	-0.476	(-0.562, -0.390)	<0.001
Baseline Proteinuria	Per unit increase	0.714	(0.577, 0.850)	<0.001
Time from Biopsy to Treatment	Per unit increase	0.059	(-0.036, 0.153)	0.220
Glucocorticoid Use	No vs. Yes (reference)	0.232	(-0.314, 0.779)	0.401
Interactions				
Treatment Group × Treatment Time	Budesonide EC × Treatment Time	0.244	(0.120, 0.367)	<0.001
Treatment Group × Baseline Proteinuria	Budesonide EC × Baseline Proteinuria	-0.275	(-0.456, -0.094)	0.003

Treatment Time: months since treatment initiation.

Model Specifications: A linear mixed-effects model was fitted to repeated measures of 24-hour proteinuria, with a random intercept for each subject and a first-order autoregressive [AR(1)] covariance structure for within-subject residuals.

Model Fit Indices: –2 Restricted Log Likelihood = 1181.197; Akaike Information Criterion (AIC) = 1187.197; Bayesian Information Criterion (BIC) = 1198.710.

The final model was selected based on the lowest AIC among candidate models (see Information Criteria panel).

**Figure 2:**
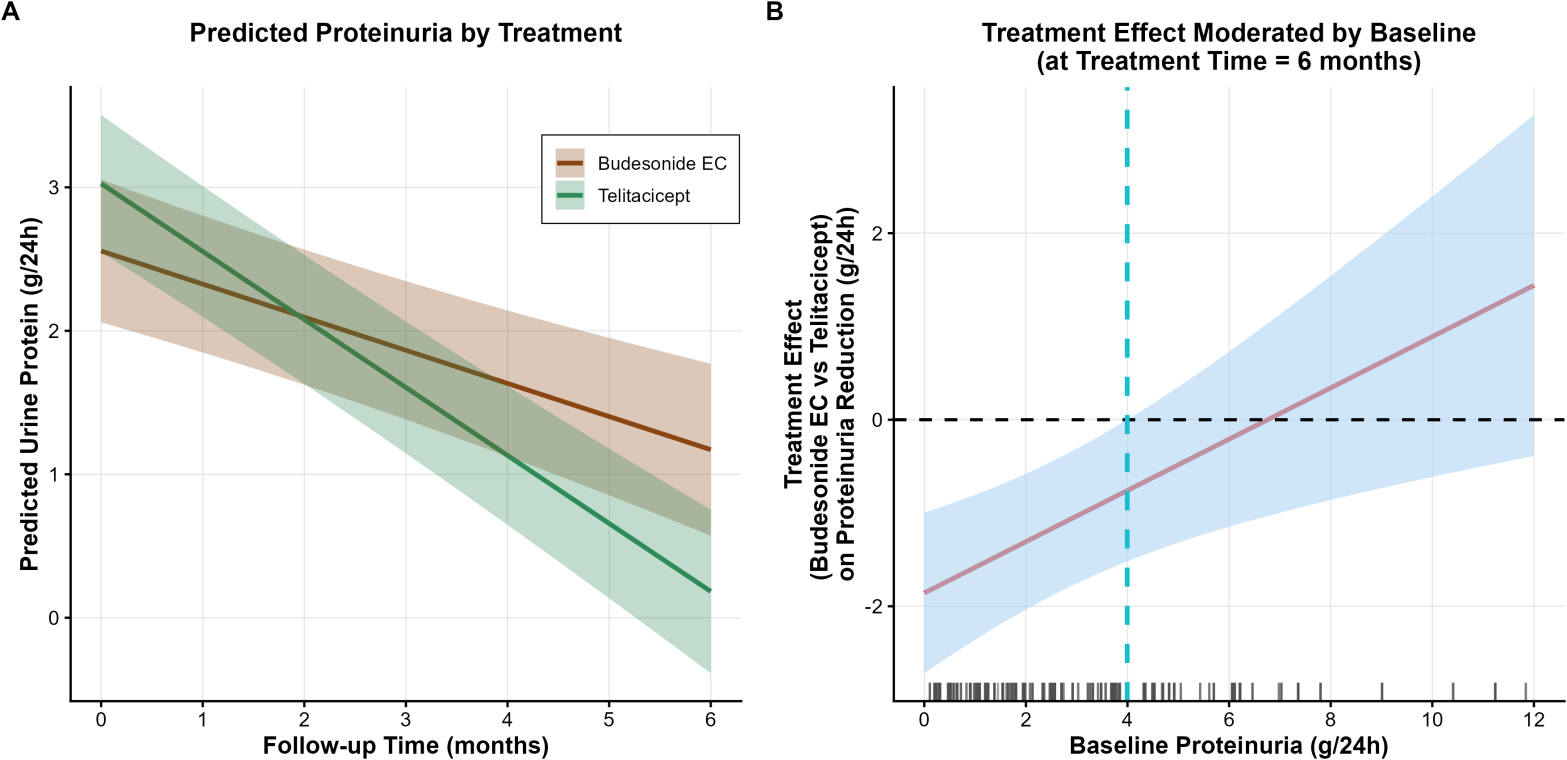
Longitudinal changes in proteinuria: effect modification by baseline proteinuria. **(A)** Observed mean proteinuria trajectories by treatment group over 6 months. Baseline proteinuria was higher in the telitacicept group (median 3.48 g/24h, IQR 1.96–4.87) than in the budesonide EC group (median 1.50 g/24h, IQR 0.65–2.58). The interaction between treatment and time was significant in the primary model but not after propensity score matching (β = –0.009, p = 0.919), indicating that the apparent difference in slopes likely reflects baseline imbalance rather than a true difference in rate of decline. **(B)** Treatment effect (budesonide EC minus telitacicept) on proteinuria reduction at 6 months, by baseline proteinuria. The vertical dashed line indicates the Johnson–Neyman–derived threshold at approximately 4 g/24h, where the 95% confidence interval crosses zero. This value is exploratory and lies near the upper limit of the overall baseline distribution (median 2.33 g/24h; IQR 1.05–4.35). Among the 95 patients, 18 of 49 (37%) in the telitacicept group and 7 of 46 (15%) in the budesonide EC group had baseline proteinuria >4 g/24h.

To explore the significant interaction between treatment group and baseline proteinuria (β = –0.275; p = 0.003), we applied the Johnson–Neyman technique. The analysis suggested that the relative benefit of telitacicept over budesonide EC in reducing proteinuria was greater at lower baseline levels, with the statistical significance of the between-group difference diminishing as baseline proteinuria increased (**Figure 2B**). This interaction remained significant after PSM (p = 0.003; **Supplementary Table 1**).

### Longitudinal changes in eGFR: association with treatment group

**Figure 3:**
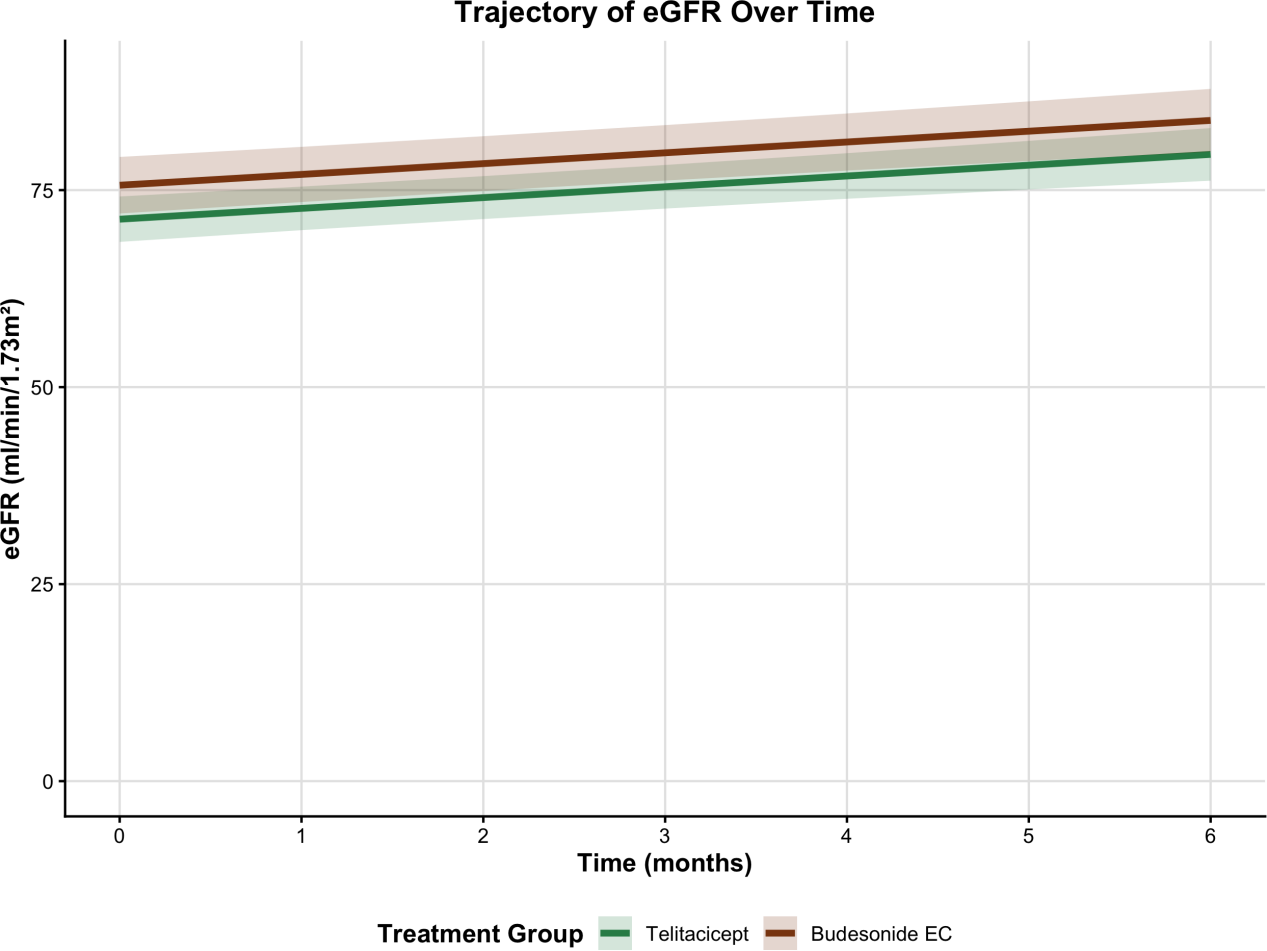
Longitudinal changes in eGFR: differential trajectories by treatment group. Estimated mean eGFR trajectories over 6 months by treatment group, derived from a linear mixed-effects model adjusted for prespecified baseline covariates. In the full cohort, budesonide EC was associated with higher eGFR during follow-up compared to telitacicept (β = 4.090; p = 0.018). This difference was attenuated and no longer statistically significant after propensity score matching (β = 5.022; p = 0.085). These trajectories reflect observed longitudinal patterns and should not be interpreted as evidence of a causal kidney-protective effect of either therapy.

Analysis of the linear mixed-effects model showed that treatment group was significantly associated with follow-up eGFR in the primary analysis adjusted for prespecified covariates, patients treated with budesonide EC had a higher mean eGFR over six months than those receiving telitacicept (β = 4.090 mL/min/1.73 m²; 95% CI, 0.720–7.459; p = 0.018; **Table 3**; **Figure 3**). Longer treatment duration (β = 1.297 per month; 95% CI, 0.764–1.829; p < 0.001) and higher baseline eGFR (β = 0.957 per unit; 95% CI, 0.913–1.001; p < 0.001) were independently associated with better eGFR trajectories.

**Table 3:** Estimated difference in longitudinal eGFR between budesonide EC and telitacicept: results from the linear mixed-effects model.

Variable	Comparison	Estimate (β)	95% CI	p-value
Fixed Effects				
Intercept		8.034	(2.207, 13.860)	0.007
Sex	Female vs. Male (reference)	-3.618	(-6.531, -0.706)	0.015
Age	Per year increase	-0.117	(-0.227, 0.008)	0.036
Treatment Group	Budesonide EC vs.Telitacicept (reference)	4.090	(0.720, 7.459)	0.018
Treatment Time	Per month	1.297	(0.764, 1.829)	<0.001
Baseline eGFR	Per mL/min/1.73m² increase	0.957	(0.913, 1.001)	<0.001
Time from Biopsy to Treatment	Per unit increase	0.042	(-0.559, 0.643)	0.890
Glucocorticoid Use	No vs. Yes (reference)	-2.858	(-6.227, -0.511)	0.095

Treatment Time: months since treatment initiation.

Model Specifications: A linear mixed-effects model was fitted to repeated measures of eGFR. The model included a random intercept for each subject and a first-order autoregressive [AR(1)] covariance structure for the within-subject residuals.

Model Fit Indices: -2 Restricted Log Likelihood = 2401.9; Akaike Information Criterion (AIC) = 2405.9; Bayesian Information Criterion (BIC) = 2413.4.

The final model was selected based on the lowest AIC among candidate models (see Information Criteria panel).

However, when baseline imbalances in age, proteinuria, glucocorticoid use, and time from biopsy to treatment were balanced via PSM (n = 48), the between-group difference in eGFR was attenuated and no longer statistically significant (β = 5.022; 95% CI, –0.731–10.776; p = 0.085; **Supplementary Table 1**).

### Sensitivity analyses

We conducted six prespecified sensitivity analyses for each outcome (**Supplementary Table 1**).

The interaction between treatment group and baseline proteinuria remained statistically significant across all scenarios, including after propensity score matching (PSM) on four covariates that differed at baseline—age, time from biopsy to treatment initiation, baseline proteinuria, and glucocorticoid use (β = –0.519; 95% CI, –0.848 – –0.192; p = 0.003; matched n = 48).

In contrast, the interaction between treatment group and treatment time for proteinuria was statistically significant in the primary model (β = 0.244; p < 0.001) but lost significance after PSM (β = –0.009; p = 0.919). Given this attenuation and the reversal in effect direction, the apparent difference in trajectory slopes between groups should not be interpreted as evidence of a differential rate of proteinuria reduction. Instead, it likely reflects baseline imbalance, as the telitacicept group had substantially higher proteinuria at baseline (**Figure 2A**).

For eGFR, the estimated association with budesonide EC remained consistent in direction across all sensitivity models (primary β = 4.090; PSM β = 5.022) but lost statistical significance after matching (95% CI, –0.731–10.776; p = 0.085). This attenuation may reflect two factors. First, the original estimate in the full cohort could have been partially inflated by baseline differences in eGFR, which were more favorable in the budesonide EC group. Second, although baseline characteristics were adequately balanced after propensity score matching, including baseline eGFR (p = 0.416 in the matched cohort), the reduced sample size likely limited statistical precision.

## Discussion

This comparative study suggests that budesonide EC and telitacicept may have distinct therapeutic profiles in IgAN, with treatment effects potentially modified by baseline disease severity. Both agents have been associated with eGFR stabilization and proteinuria reduction in prior studies (29, 30). In our head-to-head comparison, the interaction between treatment group and baseline proteinuria was statistically significant (β = –0.275; p = 0.003) and remained robust across all prespecified sensitivity analyses, including propensity score matching (p = 0.003), suggesting that telitacicept may be associated with a relatively greater antiproteinuric effect than budesonide EC at mild-to-moderate baseline proteinuria levels. By contrast, although budesonide EC was associated with a higher mean eGFR over six months in the primary analysis (p = 0.018), this difference attenuated and lost statistical significance after matching (p = 0.085), likely reflecting baseline imbalance rather than a true differential effect on renal trajectory.

Johnson–Neyman analysis identified a statistical threshold near 4.0 ;g/24 h, above which the relative antiproteinuric advantage of telitacicept over budesonide EC diminished. However, this threshold lies at the upper extreme of the observed baseline distribution, with few patients exhibiting proteinuria exceeding 6.5 ;g/24 h. The resulting sparsity limits estimation precision in the high-severity range and precludes reliable inference about treatment effects in nephrotic-range disease. Collectively, these findings indicate that the comparative antiproteinuric response varies with baseline severity, but validation in cohorts encompassing a broader spectrum of proteinuria is required before clinical application.

Both budesonide EC and telitacicept were associated with stable eGFR over six months, consistent with their known effects on disease activity in IgAN (31, 32). In the primary analysis, budesonide EC showed a nominally greater mean eGFR compared with telitacicept, though this difference lost statistical significance after PSM (p = 0.085). Given that proteinuria—a marker of active glomerular inflammation—often responds more rapidly to immunomodulation than eGFR, short-term eGFR trajectories may not fully capture the renal protective potential of either agent.

Unlike placebo-controlled trials that assess monotherapy efficacy, this study offers preliminary real-world evidence on the comparative effectiveness of two active immunomodulatory regimens in IgAN. The observation that the relative antiproteinuric benefit of telitacicept versus budesonide EC varies with baseline proteinuria lends observational support to the KDIGO 2021 framework advocating risk-adapted management (33). It generates a testable hypothesis that baseline proteinuria may inform future treatment selection between these two agents. Nevertheless, these patterns reflect associations observed in routine clinical practice and do not constitute evidence sufficient to guide individualized treatment decisions outside prospective validation.

The distinct antiproteinuric profiles of the two agents may relate to their divergent mechanisms of action. Budesonide EC targets gut-associated lymphoid tissue to reduce galactose-deficient IgA1 production, a key upstream driver in IgAN pathogenesis (31). This approach was associated with proteinuria reduction in the NEFIGAN trial (18). Telitacicept inhibits B-cell survival through dual blockade of BLyS and APRIL, thereby suppressing autoantibody generation (34, 35), which also translates into antiproteinuric effects. The observation that telitacicept’s relative benefit varies by baseline proteinuria may reflect underlying heterogeneity in disease activity, as B-cell–mediated pathways have been linked to disease severity in prior studies (36–38). However, because immunological biomarkers were not measured, this study cannot evaluate whether the observed effect modification reflects underlying differences in B-cell activity.

Despite the use of linear mixed-effects models, pre-specified interaction testing, Johnson–Neyman analysis, multivariable adjustment, and PSM, the inherent constraints of a retrospective design persist. Concomitant glucocorticoid use differed substantially between groups (51.0% with telitacicept versus 10.9% with budesonide EC), introducing a major source of residual confounding that statistical adjustment cannot fully resolve. This imbalance likely explains the attenuation of the eGFR association after matching (p = 0.085). In contrast, the interaction of treatment group and baseline proteinuria remained consistent across all analytic strategies (p = 0.003), indicating relative insensitivity to this confounder. The observed antiproteinuric difference therefore reflects the net effect of each real-world treatment regimen rather than the isolated effect of telitacicept. Isolating the specific contribution of telitacicept requires randomized trials with standardized background immunosuppression.

Beyond medication use, the availability of standardized histopathological assessment represents another key dimension in real-world treatment selection. Although all patients had biopsy-confirmed IgAN, formal application of the Oxford-MEST-C classification was feasible in only 76 patients. Within this subgroup, the distribution of histological lesions did not differ meaningfully between treatment groups (p = 0.646), implying that initial therapy selection was shaped predominantly by clinical factors rather than standardized pathological features. Because standardized lesion scoring was unavailable for a substantial proportion of patients, we were unable to assess whether specific histological features such as endocapillary hypercellularity or cellular crescents influence treatment response to targeted immunomodulatory therapies. This requires prospective trials with protocol-specified kidney biopsy evaluation.

Collectively, these findings indicate that baseline proteinuria modifies the comparative antiproteinuric effect of telitacicept versus budesonide EC in IgAN, with a relatively greater benefit of telitacicept observed at mild-to-moderate levels. This pattern aligns with the KDIGO 2021 recommendation for risk-adapted management and persisted across all prespecified sensitivity analyses. Nevertheless, as an observational study subject to residual confounding and selection bias, our results support a testable hypothesis that baseline severity may inform future treatment selection rather than provide evidence sufficient to guide individualized therapy. Prospective validation in diverse populations is essential before clinical implementation.

### Several limitations should be acknowledged

First, as with all observational studies, our findings are susceptible to residual confounding from unmeasured or imperfectly measured factors. Although sensitivity analyses—including PSM—support the robustness of effect modification by baseline proteinuria, the results reflect associations observed in real-world clinical practice and do not establish causality; accordingly, all conclusions should be interpreted with caution.

Second, our findings derive from a highly selected subgroup: of 381 individuals initially identified, only 95 fulfilled all inclusion criteria. This reflects real-world data limitations, including absent longitudinal measurements, inconsistent treatment adherence, and protocol deviations such as therapy switching. As shown in **Figure 1**, excluded patients were more likely to have incomplete clinical documentation or unstable disease trajectories. Consequently, the results may not generalize to unselected IgAN populations, particularly those with suboptimal adherence or fragmented follow-up.

Third, standardized Oxford-MEST-C scoring was unavailable for 19 patients (20%) due to non-structured historical pathology reports. As a result, histological lesion burden could not be incorporated into the primary analytical models, limiting adjustment for baseline renal structural severity.

Fourth, although 25 of 95 patients (26%) had baseline proteinuria above 4 ;g/24h, the overall distribution was skewed toward lower values, and very few exceeded 6.5 ;g/24h. This limits precision at the upper severity range and precludes definitive conclusions about treatment effects in severe disease.

Fifth, nearly all patients initiated treatment within six months of biopsy-proven diagnosis, reflecting a focus on early intervention. This may limit generalizability to individuals with long-standing or advanced IgAN, in whom structural kidney damage may diminish responsiveness to immunomodulation.

Sixth, the single-center setting may affect generalizability across diverse healthcare systems.

Finally, the 6-month follow-up is too short to assess hard renal endpoints.

## Conclusions

This real-world head-to-head comparison of budesonide EC and telitacicept in IgAN suggests that baseline proteinuria may modify their relative antiproteinuric effects. Telitacicept was associated with a greater reduction in proteinuria than budesonide EC among patients with mild-to-moderate baseline levels, with a statistically significant interaction of treatment group and baseline proteinuria that persisted after PSM. Johnson–Neyman analysis identified a threshold near 4.0 ;g/24h, though sparse data above this level limit interpretability in severe disease.

In contrast, the higher mean eGFR observed with budesonide EC over six months in the primary analysis attenuated after matching and likely reflects baseline imbalance rather than a differential effect on renal trajectory.

These findings are hypothesis-generating and do not support clinical guidance. Prospective trials are needed to determine whether baseline proteinuria can inform therapy selection between these agents.

## Data Availability

The original contributions presented in the study are included in the article/**Supplementary Material**. Further inquiries can be directed to the corresponding author.

## Supplementary material

Supplementary Table 1 Sensitivity analyses for the primary mixed-effects models.

Supplementary Table 2 Oxford-MEST-C histopathology scores in the subgroup with available biopsy data (n = 76).

## References

[1] Kidney Disease: Improving Global Outcomes (KDIGO) IgAN and IgAV Work Group . KDIGO 2025 clinical practice guideline for the management of immunoglobulin A nephropathy (IgAN) and immunoglobulin A vasculitis (IgAV). Kidney Int. (2025) 108:S1–S71. doi: 10.1016/j.kint.2025.04.004 40975564

[2] GBD 2023 Cancer Collaborators . The global, regional, and national burden of cancer, 1990-2023, with forecasts to 2050: a systematic analysis for the Global Burden of Disease Study 2023. Lancet. (2025) 406:1565–86. doi: 10.1016/S0140-6736(25)01635-6. PMID: 41015051 PMC12687902

[3] Roberts ISD . Pathology of IgA nephropathy: a global perspective. Nephrol (Carlton). (2024) 29:71–4. doi: 10.1111/nep.14343. PMID: 39327761

[4] Lai KN Leung JC Tang SC . Recent advances in the understanding and management of IgA nephropathy. F1000Res. (2016) 5:F1000 Faculty Rev-161. doi: 10.12688/f1000research.7352.1. PMID: 26918170 PMC4755398

[5] Shen X Chen P Liu M Liu L Shi S Zhou X . Long-term outcomes of IgA nephropathy in China. Nephrol Dialysis Transplant. (2025) 40:1137–46. doi: 10.1093/ndt/gfae252. PMID: 39557651

[6] Pitcher D Braddon F Hendry B Mercer A Osmaston K Saleem MA . Long-term outcomes in IgA nephropathy. Clin J Am Soc Nephrol. (2023) 18:727–38. doi: 10.2215/CJN.0000000000000135. PMID: 37055195 PMC10278810

[7] Lv J Wong MG Hladunewich MA Jha V Hooi LS Monaghan H . Effect of oral methylprednisolone on decline in kidney function or kidney failure in patients with IgA nephropathy: the TESTING randomized clinical trial. JAMA. (2022) 327:1888–98. doi: 10.1001/jama.2022.5368. PMID: 35579642 PMC9115617

[8] Sugiura N Moriyama T Miyabe Y Karasawa K Nitta K . Severity of arterial and/or arteriolar sclerosis in IgA nephropathy and the effects of renin-angiotensin system inhibitors on its prognosis. J Pathol Clin Res. (2021) 7:616–23. doi: 10.1002/cjp2.234. PMID: 34185389 PMC8503890

[9] Kim J Kamal F Lafayette R . Current trial landscape of IgA nephropathy therapy. Med. (2026) 7:100940. doi: 10.1016/j.medj.2025.100940. PMID: 41519110

[10] Lafayette R Kristensen J Stone A Floege J Tesař V Trimarchi H . Efficacy and safety of a targeted-release formulation of budesonide in patients with primary IgA nephropathy (NefIgArd): 2-year results from a randomised phase 3 trial. Lancet. (2023) 402:859–70. doi: 10.1016/S0140-6736(23)01554-4. PMID: 37591292

[11] Barratt J Rovin BH Cattran D Floege J Lafayette R Tesar V . Why target the gut to treat IgA nephropathy? Kidney Int Rep. (2020) 5:1620–4. doi: 10.1016/j.ekir.2020.08.009. PMID: 33102954 PMC7569689

[12] Barratt J Lafayette R Kristensen J Stone A Cattran D Floege J . Results from part A of the multi-center, double-blind, randomized, placebo-controlled NefIgArd trial, which evaluated targeted-release formulation of budesonide for the treatment of primary immunoglobulin A nephropathy. Kidney Int. (2023) 103:391–402. doi: 10.1016/j.kint.2022.09.017. PMID: 36270561

[13] Fellström BC Barratt J Cook H Coppo R Feehally J de Fijter JW . Targeted-release budesonide versus placebo in patients with IgA nephropathy (NEFIGAN): a double-blind, randomised, placebo-controlled phase 2b trial. Lancet. (2017) 389(10084):2117–2127. doi: 10.1016/S0140-6736(17)30550-0 28363480

[14] Lv J Liu L Hao C Li G Fu P Xing G . Randomized phase 2 trial of telitacicept in patients with IgA nephropathy with persistent proteinuria. Kidney Int Rep. (2023) 8:499–506. doi: 10.1016/j.ekir.2022.12.014. PMID: 36938094 PMC10014376

[15] Kim D Neuen BL Perkovic V Wong MG . Effects of therapies on proteinuria and eGFR in IgA nephropathy: meta-analysis of randomized trials. Clin J Am Soc Nephrol. (2025) 20:1753–66. doi: 10.2215/CJN.0000000839. PMID: 40928891 PMC12708380

[16] Ştefan G Stancu S Zugravu A Petre N . Comparing long-term outcomes in glomerular disease patients presenting with nephrotic syndrome versus nephrotic range proteinuria. Life (Basel). (2024) 14:1674. doi: 10.3390/life14121674. PMID: 39768381 PMC11728368

[17] Heerspink HJL Rovin BH Komers R Hendry B Mercer A Preciado P . Association between complete proteinuria remission and kidney function in the phase 3 PROTECT trial of sparsentan in IgA nephropathy. Clin J Am Soc Nephrol. (2026) 21(4):578–592. doi: 10.2215/CJN.0000000961. PMID: 41428405 PMC13065159

[18] Stamellou E Seikrit C Tang SCW Boor P Tesař V Floege J . IgA nephropathy. Nat Rev Dis Primers. (2023) 9:67. doi: 10.1038/s41572-023-00476-9. PMID: 38036542

[19] Zachova K Jemelkova J Kosztyu P Ohyama Y Takahashi K Zadrazil J . Galactose-deficient IgA1 B cells in the circulation of IgA nephropathy patients carry preferentially lambda light chains and mucosal homing receptors. J Am Soc Nephrol. (2022) 33:908–17. doi: 10.1681/ASN.2021081086. PMID: 35115327 PMC9063893

[20] Sridharan K Sivaramakrishnan G . Drug therapies for patients with IgA nephropathy: a network meta-analysis of randomized clinical trials. Curr Clin Pharmacol. (2020) 15:132–44. doi: 10.2174/1574884715666191223103914. PMID: 31870272

[21] Yamaguchi Y Kosugi T Sasaki T Haruhara K Okabayashi Y Okabe M . The association between low-grade proteinuria and adverse kidney outcomes in IgA nephropathy: a systematic review and meta-analysis. Clin J Am Soc Nephrol. (2026) 21(4):615–625. doi: 10.2215/CJN.0000000952. PMID: 41385286 PMC13065120

[22] Li R Hui M Chen P Zhang D Tang C Zhou X . The association of proteinuria target achievement timing and stability with adverse kidney outcomes among patients with IgA nephropathy: a cohort study. Am J Nephrol. (2025) 6:1–17. doi: 10.1159/000547868. PMID: 40769132

[23] Jongs N Heerspink HJL . What are the right endpoints to assess the effectiveness of new treatments for IgAN?. Nephrol Dial Transplant. (2026) 41(Supplement_1):i46–i52. doi: 10.1093/ndt/gfaf184. PMID: 40985592

[24] Kim D Neuen BL Perkovic V Wong MG . Effects of therapies on proteinuria and eGFR in IgA nephropathy: meta-analysis of randomized trials. Clin J Am Soc Nephrol. (2025) 20:1753–66. doi: 10.2215/CJN.0000000839. PMID: 40928891 PMC12708380

[25] Yau K Reich HN . How should we measure and interpret glomerular inflammation and what is the best anti-inflammatory approach in IgA nephropathy?. Nephrol Dial Transplant. (2026) 41(Supplement_1):i15–i26. doi: 10.1093/ndt/gfaf135. PMID: 40795024

[26] Lorenzi M Ali SN Cadarette S Ebohon S Diaby V Mathur M . Association between surrogate endpoints and clinical outcomes in immunoglobulin A nephropathy: a systematic literature review. Adv Ther. (2025) 42:4824–65. doi: 10.1007/s12325-025-03331-3. PMID: 40828351 PMC12474693

[27] Levey AS Stevens LA Schmid CH Zhang YL Castro A Feldman HI . A new equation to estimate glomerular filtration rate. Ann Intern Med. (2009) 150:604–12. doi: 10.7326/0003-4819-150-9-200905050-00006. PMID: 19414839 PMC2763564

[28] Aubin AMAGNIDE Micheline GBEHA Romain GK . Fitting an optimal variance-covariance structure for longitudinal data under linear mixed effects models framework: simulation based analysis. Afr J Appl Stat. (2018) 5:489–502. doi: 10.16929/ajas/489.226. PMID: 41130871

[29] Zhang H Lafayette R Wang B Ying L Zhu Z Stone A . Efficacy and safety of nefecon in patients with IgA nephropathy from Mainland China: 2-year NefIgArd trial results. Kidney360. (2024) 5:1881–92. doi: 10.34067/KID.0000000583. PMID: 39724565 PMC11687989

[30] Barratt J Stone AM Reich HN Lafayette RA . eGFR slope modelling predicts long-term clinical benefit with nefecon in a real-world IgAN population. Clin Kidney J. (2024) 18:sfae404. doi: 10.1093/ckj/sfae404. PMID: 40235631 PMC11997755

[31] Heerspink HJL Rovin BH Komers R Hendry B Mercer A Preciado P . Association between complete proteinuria remission and kidney function in the phase 3 PROTECT trial of sparsentan in IgA nephropathy. Clin J Am Soc Nephrol. (2026) 21(4):578–592. doi: 10.2215/CJN.0000000961. PMID: 41428405 PMC13065159

[32] Shu H Wang Y Li X Zhang L Wei Z Song Y . Telitacicept plus low-dose mycophenolate mofetil in the treatment of IgA nephropathy: a retrospective study. Clin Exp Med. (2025) 25:287. doi: 10.1007/s10238-025-01829-2. PMID: 40788583 PMC12339650

[33] Kidney Disease: Improving Global Outcomes (KDIGO) Glomerular Diseases Work Group . KDIGO 2021 clinical practice guideline for the management of glomerular diseases. Kidney Int. (2021) 100:S1–S276. doi: 10.1016/j.kint.2021.05.021. PMID: 34556256

[34] Lafayette R Barbour SJ Brenner RM Campbell KN Doan T Eren N . A phase 3 trial of atacicept in patients with IgA nephropathy. N Engl J Med. (2026) 394(7):647–657. doi: 10.1056/NEJMoa2510198. PMID: 41196369

[35] Perkovic V Trimarchi H Tesař V Lafayette R Wong MG Barratt J . Sibeprenlimab in IgA nephropathy - interim analysis of a phase 3 trial. N Engl J Med. (2026) 394(7):635–646. doi: 10.1056/NEJMoa2512133. PMID: 41211929

[36] Roberts L Barratt J . Targeting B cells in IgA nephropathy: from pathogenic insight to therapeutic horizon. Clin Kidney J. (2025) 18:ii26–34. doi: 10.1093/ckj/sfaf322. PMID: 41393859 PMC12699742

[37] Moszczuk B Mucha K Kucharczyk R Zagożdżon R . The role of glomerular and serum expression of lymphocyte activating factors BAFF and APRIL in patient with membranous and IgA nephropathies. Arch Immunol Ther Exp (Warsz). (2025) 73(1). doi: 10.2478/aite-2025-0018 40472229

[38] Sallustio F Curci C Chaoul N Fontò G Lauriero G Picerno A . High levels of gut-homing immunoglobulin A+ B lymphocytes support the pathogenic role of intestinal mucosal hyperresponsiveness in immunoglobulin A nephropathy patients. Nephrol Dial Transplant. (2021) 36:452–64. doi: 10.1093/ndt/gfaa264. PMID: 33200215 PMC7898021

