# Supplementary material for: Comparative effectiveness of budesonide EC and telitacicept in proteinuria and eGFR trajectories in IgA nephropathy: a retrospective cohort study
Source: Front Immunol. 2026 May 13;17:1821774. doi: 10.3389/fimmu.2026.1821774 (PMC13212180; doi:10.3389/fimmu.2026.1821774)
Supplement: Supplementary Table 1 — Sensitivity analyses for the primary mixed-effects models. [file Table1.doc]

| **Supplementary Table 1. Sensitivity Analyses for the Primary Mixed-Effects Models** | | | | | |
| --- | --- | --- | --- | --- | --- |
| **Analysis (Model)** | **Key Parameter** | **Estimate (β)** | **95% CI** | **p-value** | **Conclusion** |
| **A. Proteinuria Model** |  |  |  |  |  |
| **SA (PRO): Primary Model (Table 2)** | **Treatment Group × Treatment Time（month）** | **0.244** | **(0.120, 0.367)** | **<0.001** | **Reference** |
| SA-1 (PRO):(-age, -glucocorticoid use, -time from biopsy) | Treatment Group × Treatment Time（month） | 0.247 | (0.123, 0.370) | <0.001 | Robust |
| SA-2 (PRO): (+ baseline egfr, hypertension, diabetes) | Treatment Group × Treatment Time（month） | 0.243 | (0.119, 0.367) | <0.001 | Robust |
| SA-3 (PRO): (+ all covariates) | Treatment Group × Treatment Time（month） | 0.242 | (0.118, 0.366) | <0.001 | Robust |
| SA-4 (PRO): (Exclude outliers) | Treatment Group × Treatment Time（month） | 0.291 | (0.187, 0.395) | ＜0.001 | Robust |
| SA-5 (PRO): (Using CS covariance structure) | Treatment Group × Treatment Time（month） | 0.251 | (0.128, 0.356) | ＜0.001 | Robust |
| SA-6 (PRO): (PSM on 4 imbalanced covariates) | Treatment Group × Treatment Time（month） | -0.009 | (-0.187,0.168) | 0.919 | Not robust |
| **SA (PRO): Primary Model (Table 2)** | **Treatment Group × Baseline Proteinuria(g/24h)** | **-0.275** | **(-0.456, -0.094)** | **0.003** | **Reference** |
| SA-1 (PRO):(-age, -glucocorticoid use, -time from biopsy) | Treatment Group × Baseline Proteinuria(g/24h) | -0.243 | (-0.416,-0.070) | 0.006 | Robust |
| SA-2 (PRO):(+ baseline egfr,hypertension,diabetes) | Treatment Group × Baseline Proteinuria(g/24h) | -0.221 | (-0.400,-0.042) | 0.016 | Robust |
| SA-3 (PRO): (+ all covariates) | Treatment Group × Baseline Proteinuria(g/24h) | -0.235 | (-0.420, -0.051) | 0.013 | Robust |
| SA-4 (PRO): (Exclude outliers) | Treatment Group × Baseline Proteinuria(g/24h) | -0.348 | (-0.523, -0.173) | ＜0.001 | Robust |
| SA-5 (PRO):(Using CS covariance structure) | Treatment Group × Baseline Proteinuria(g/24h) | -0.274 | (-0.465, -0.082) | 0.006 | Robust |
| SA-6 (PRO): (PSM on 4 imbalanced covariates) | Treatment Group × Baseline Proteinuria(g/24h) | -0.519 | (-0.848, -0.192) | 0.003 | Robust |
| **B. eGFR Model** |  |  |  |  |  |
| **SA (eGFR):Primary (Table 3)** | **Treatment Group (Budesonide EC vs Telitacicept)** | **4.090** | **(0.072, 7.459)** | **0.018** | **Reference** |
| SA-1 (eGFR):(-glucocorticoid use, -time from biopsy) | Treatment Group (Budesonide EC vs Telitacicept) | 3.122 | (0.153, 6.090) | 0.039 | Robust |
| SA-2 (eGFR):(+ baseline proteinuria,hypertension,diabetes ) | Treatment Group (Budesonide EC vs Telitacicept) | 4.437 | (0.987, 7.888) | 0.012 | Robust |
| SA-3 (eGFR): (+ all covariates) | Treatment Group (Budesonide EC vs Telitacicept) | 4.446 | (1.045, 7.848) | 0.011 | Robust |
| SA-4 (eGFR): (Exclude outliers) | Treatment Group (Budesonide EC vs Telitacicept) | 4.113 | (0.939, 7.287) | 0.012 | Robust |
| SA-5 (eGFR):(Using CS covariance structure) | Treatment Group (Budesonide EC vs Telitacicept) | 4.349 | (1.447, 7.961) | 0.031 | Robust |
| SA-6 (eGFR): (PSM on 4 imbalanced covariates) | Treatment Group (Budesonide EC vs Telitacicept) | 5.022 | (-0.731, 10.776) | 0.085 | Not robust |

SA (PRO): Primary Model for proteinuria.

SA-1 (PRO): Excluding covariates imbalanced at baseline.

SA-2 (PRO): Adjustment for baseline renal parameters and comorbidities.

SA-3 (PRO): Including all measured baseline variables.

SA-4 (PRO): Exclusion of patients with extreme standardized residuals (|ZRE| > 3).

SA-5 (PRO): Re-estimation using a compound symmetry covariance structure.

SA-6 (PRO): PSM on 4 imbalanced covariates (age, time from biopsy to treatment, baseline proteinuria,

glucocorticoid use; all P < 0.05 in Table 1, n=48), Baseline eGFR did not differ significantly (P = 0.416).

SA (eGFR): Primary Model for eGFR.

SA-1 (eGFR): Excluding covariates imbalanced at baseline.

SA-2 (eGFR): Adjustment for baseline renal parameters and comorbidities.

SA-3 (eGFR): Including all measured baseline variables.

SA-4 (eGFR): Exclusion of patients with extreme standardized residuals (|ZRE| > 3).

SA-5 (eGFR): Re-estimation using a compound symmetry covariance structure.

SA-6 (eGFR): PSM on 4 imbalanced covariates (age, time from biopsy to treatment, baseline proteinuria,

glucocorticoid use; all P < 0.05 in Table 1, n=48), Baseline eGFR did not differ significantly (P = 0.416).
