# Supplementary material for: Comparative effectiveness of budesonide EC and telitacicept in proteinuria and eGFR trajectories in IgA nephropathy: a retrospective cohort study
Source: Front Immunol. 2026 May 13;17:1821774. doi: 10.3389/fimmu.2026.1821774 (PMC13212180; doi:10.3389/fimmu.2026.1821774)
Supplement: Supplementary Table 2 — Oxford-MEST-C histopathology scores in the subgroup with available biopsy data (n = 76). [file Table2.doc]

| Supplementary Table 2. Oxford-MEST-C Histopathology Scores in the Subgroup with Available Biopsy Data (n = 76) | | | |
| --- | --- | --- | --- |
| **Variable** | **Budesonide EC (n = 35)** | **Telitacicept (n = 41)** | **p-value** |
| MEST-C total score, median | 2.0 (2.0–4.0) | 3.0 (2.0–3.5) | 0.632 |
| M0, n (%) | 19 (54.3%) | 20 (48.8%) | 0.653 |
| E0, n (%) | 17 (48.6%) | 27 (65.9%) | 0.128 |
| S0, n (%) | 7 (20.0%) | 6 (14.6%) | 0.536 |
| T0, n (%) | 26 (56.5%) | 26 (53.0%) | 0.386 |
| C0, n (%) | 21 (45.6%) | 22 (44.8%) | 0.572 |
| Among 95 biopsy-proven IgA nephropathy cases, MEST-C scores were extractable from pathology reports in 76 (80%); the rest lacked sufficient detail for full scoring. P-values: Mann–Whitney U test (total score), Fisher’s exact test (components). | | | |
